# Photoinhibition of *Phaeocystis globosa* resulting from oxidative stress induced by a marine algicidal bacterium *Bacillus* sp. LP-10

**DOI:** 10.1038/srep17002

**Published:** 2015-11-25

**Authors:** Chengwei Guan, Xiaoyun Guo, Yi Li, Huajun Zhang, Xueqian Lei, Guanjing Cai, Jiajia Guo, Zhiming Yu, Tianling Zheng

**Affiliations:** 1 State Key Laboratory of Marine Environmental Science and Key Laboratory of Ministry of Education for Coastal and Wetland Ecosystems, School of Life Sciences, Xiamen University, Xiamen 361102, China; 2 Tobacco Science Institute of Jiangxi Province, Nanchang 330025, China; 3 School of Chemistry and Chemical Engineering, Xiamen University, Xiamen 361102, China; 4 College of Life Sciences, Henan Normal University, Xinxiang 453007, China; 5 Key Laboratory of Marine Ecology and Environmental Science, Institute of Oceanology, Chinese Academy of Sciences, Qingdao 266071, China

## Abstract

Harmful algal blooms caused by *Phaeocystis globosa* have resulted in staggering losses to coastal countries because of their world-wide distribution. Bacteria have been studied for years to control the blooms of harmful alga, however, the action mechanism of them against harmful algal cells is still not well defined. Here, a previously isolated algicidal bacterium *Bacillus* sp. LP-10 was used to elucidate the potential mechanism involved in the dysfunction of *P. globosa* algal cells at physiological and molecular levels. Our results showed *Bacillus* sp. LP-10 induced an obvious rise of reactive oxygen species (ROS), which was supposed to be major reason for algal cell death. Meanwhile, the results revealed a significant decrease of photosynthetic physiological indexes and apparent down-regulated of photosynthesis-related genes (*psb*A and *rbcS*) and protein (PSII reaction center protein D1), after treated by *Bacillus* sp. LP-10 filtrates, suggesting photoinhibition occurred in the algal cells. Furthermore, our results indicated that light played important roles in the algal cell death. Our work demonstrated that the major lethal reason of *P. globosa* cells treated by the algicidal bacterium was the photoinhibition resulted from oxidative stress induced by *Bacillus* sp. LP-10.

Microalgae play an important role in carbon sink during the transportation of the carbon to deep waters. However, many of them lead to harmful algal blooms (HABs), causing adverse effects on marine ecosystems and economics^1^,^2^, even harm people by producing toxins^3^. Many methods were developed to manage the HABs^4^,^5^. However, physical and chemical methods are not supposed to be optimal choices in controlling HABs, because of their obvious defects of high costs and unavailability for large-scale application, and more importantly, bringing about secondary pollution. Hence, biological methods are regarded as more promising approaches to regulate HABs^6^,^7^.

Marine bacteria are important in regulating the growth dynamics of microalga^8^,^9^. Among them, a group of bacteria, now called algicidal bacteria, are suggested to show inhibitory effects on algal growth, which are considered as excellent candidates to control HABs^10^. The action mode of algicidal bacteria on target algal cells were divided into two types, one is lysing the algal cells by contacting with the cells surface directly, named direct attack mode, the other is lysing the algal cells by releasing active substances/allelochemicals, named indirect attack mode^11^,^12^. Most algicidal bacteria reported so far belongs to the latter action mode^13^,^14^.

Despite a lot of algicidal bacteria were isolated, the action mechanisms of them against harmful algal cells were still not well defined. Recently, some researches revealed that reactive oxygen species (ROS) played significant roles in the inhibitory effects of algicidal bacteria on algal growth^15^,^16^. Some allelochemicals were documented to trigger the producing of ROS, leading to algal cells damage^17^,^18^.

As is known, environmental stresses usually induce algal cell to produce ROS, which change the oxidative level and breaks the redox balance of the algal cells^19^,^20^. Normally, antioxidant systems of cells, including superoxide dismutase (SOD), peroxidase (POD), catalase (CAT) and glutathione (GSH) etc., could protect themselves from oxidative damage by clearing the numerous ROS to keep cells at a stable redox level^21^,^22^.

Photosynthetic systems are fundamental for the growth of plant and algal cells. Generally, performing oxygenic photosynthesis will cause damage to the photosynthetic apparatus^23^. The reaction center of photosystem II (PSII) was one of the main damage target sites, of which the protein D1 is sensitive and vulnerable to stresses^24^. Photosynthetic organisms execute self-repair program to replace the impaired protein D1 via *de novo* synthesis. Normally, the protein D1 content is in a dynamic balance. However, when the protein D1 damage rate exceeds the repair rate, photoinhibition occurs in photosynthetic organisms^24^. Recent research revealed high light induced lower expression of protein D1 in diatom *Phaeodactylum tricornutum* than low light exposure^25^. Recent researches showed that environmental factors, such as NaCl, heat and low temperature can also lead to photoinhibition by interrupting the *de novo* synthesis of protein D1^23^,^26^.

*Phaeocystis globosa* Scherffel (Prymnesiophyceae) is a HABs causing species with world-wide distribution, which caused huge economic loses to coastal countries^27^. *Bacillus* sp. LP-10, a marine bacterium obtained in our laboratory before, was proved to be effective in controlling the growth of *P. globosa*^28^. However, few work were performed to illustrate the action mode of the bacterium on *P. globosa*. To better understand the action mechanism of the algicidal mechanism, a systematic investigation of physiological and molecular alterations in algal cells was carried out. And the physiological data coupled with transcript and protein analysis provided new insights into the algicidal mechanism of the marine bacterium on target algal cells.

## Materials and Methods

### Strains and culture conditions

Cultures of *P. globosa* PG03 were supplied by the State Key Laboratory of Marine Environmental Sciences, Xiamen University, China and f/2 (-Si) medium was used to prepare algal cultures for the experiments^29^. The light conditions were set to a 12 h/12 h light-dark cycle under cool-white fluorescent light with an intensity of 50 μmol photons m^−2^s^−1^ at 20 ± 1 °C.

The *Bacillus* sp. LP-10 strain was previously isolated in surface water samples from the East China Sea in our laboratory, and the GenBank accession number is KF411459. The marine bacterial strain was prepared in autoclaved Zobell 2216E medium and cultured at 28 °C for 48 h with 150 rpm rotation speed^30^. The bacterium cultures at stationary phase were collected and centrifuged at 8000 g for 10 min. The cell-free filtrates of *Bacillus* sp. LP-10 were prepared by filtering the supernatant through 0.22 μm size pore Millipore membrane filters. The cell-free filtrates of *Bacillus* sp. LP-10 was used in the whole study unless specially declaration. To ensure that the *Bacillus* sp. LP-10 is safe for the environment, the toxicity of the strain were tested. The test results indicated *Bacillus* sp. LP-10 were environmentally friendly to the aquatic environment (Supplementary Information).

### ROS assays

The fluorescence probe 2′, 7′-dichlorofluorescein diacetate (DCFH-DA) is used to measure the ROS level in algal cells treated with cell-free filtrates of *Bacillus* sp. LP-10^31^. The fluorescence intensity was measured at an excitation wavelength of 488 nm and an emission wavelength of 525 nm. For ROS detection, 20 mL algal cultures treated with strain LP-10 filtrates were centrifuged at 5000 g for 5 min, the pellets were washed twice with sterile f/2 medium, then the algal cells were incubated with DCFH-DA (final concentration 10 μM) at 37 °C for 30 min in the dark. The superfluous DCFH-DA probe was removed by centrifugation and the pellets were re-suspended in 500 μL sterile f/2 medium after washing twice. The fluorescence intensity was measured using a microplate reader and the fluorescence photos of 5% LP-10 treatment was taken by an epifluorescence microscope.

N-acetylcysteine (NAC), a common ROS scavenger, was used to clear the ROS in algal cells. 20 mL algal cultures were processed using four treatments respectively: (1) 5% (v/v) strain LP-10 filtrates (LP treatment); (2) 5% filtrates and NAC (final concentration 1 mM) (NAC + LP treatment); (3) NAC (final concentration 1 mM) (NAC control); (4) blank 2216E broth (5%) (CK). The autofluorescence of the algal cells was measured in 96-well microplates at an excitation wavelength of 440 nm and an emission wavelength of 680 nm. Similar operations were performed with glutathione (GSH) and ascorbic acid (AsA), two other ROS scavengers. The ROS contents were determined at 0, 4, 8, 12, 24 h after addition of ROS scavengers.

### The MDA content and antioxidant activities assays

Algal cultures of *P. globosa* were treated by cell-free filtrates of *Bacillus* sp. LP-10 in 3, 4, 5% concentration, followed by sampling at different time course with an interval of 12 h. Algal cells were collected at 5000 g for 5 min, and the pellets were washed twice with sterile seawater. The cells were then re-suspended in 1 mL PBS solution (0.1 M, pH 7.4), followed by sonication at 100 W for 60 times. The cell debris was removed by centrifugation at 12000 g for 10 min, and the supernatants were transferred to new Eppendorf tubes for malondialdehyde (MDA) and antioxidants analysis. The MDA content and activities of the antioxidants, including superoxide dismutase (SOD), catalase (CAT), peroxidase (POD) and GSH, were measured using MDA kits and corresponding antioxidant assay kits (Nanjing Jiancheng Bioengineering Institute) following the manufacturer’s instructions. The contents of MDA and antioxidant were expressed on a protein basis.

### Chlorophyll contents and variable chlorophyll fluorescence analysis

Chlorophyll contents were measured based on previously described methods with slight modifications^32^. 20 mL axenic exponential phase cell cultures of *P. globosa* were collected at 5000 g for 10 min and the pellets were re-suspended in 5 mL 90% ethanol overnight to extract the chlorophyll. After extraction, the pellets were removed using centrifugation, the optical density of the ethanol extract was measured at wavelengths of 645 and 664 nm using a VARY-50 spectrophotometer. The contents of chlorophyll *a* (Chl *a*) , chlorophyll *b* (Chl *b*), and the ratio of these two pigments were calculated as follows:


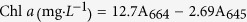



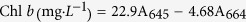



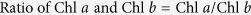


where A_645_ and A_664_ were the optical densities of ethanol extracts of algal cells at wavelengths of 645 and 664 nm.

The variable chlorophyll fluorescence was used as a proxy for the photosynthetic efficiency and capacity of the algal cell. And the variable chlorophyll fluorescence was measured using a PAM-CONTROL fluorometer (Walz, Effeltrich, Germany). The maximum quantum yield (Fv/Fm) and the maximum relative electron transport rate (rETR) were determined based on previously described methods after the algal cells were dark-adapted for 15 min^33^.

### Light condition assays

20 mL algal cultures inoculated with 5% *Bacillus* sp. LP-10 filtrates, were respectivly placed under different light conditions: the normal light intensity (normal light, 50 μmol photons m^−2^s^−1^), low light intensity (low light, 20 μmol photons m^−2^s^−1^) and the dark condition (dark). The autofluorescence of the algal cells was measured in 96-well microplates every 24 h at an excitation wavelength of 440 nm and an emission wavelength of 680 nm. The algal cultures without LP-10 filtrates under different light conditions were set up as control.

### Gene transcription analysis

For total RNA extraction, 40 mL algal cultures were treated for 24 h and harvested at 5000 g for 10 min, then the pellets were quickly frozen in liquid nitrogen and homogenized followed by addition of 1 mL RNAiso reagent (TaKaRa Biochemicals, China). The total RNA was extracted following the manufacturer’s instructions. Reverse transcription of RNA was performed using the PrimeScript™ RT-PCR Kit (TaKaRa Biochemicals, China). Two photosynthesis genes (*psbA* and *rbcS*) were selected for real-time qPCR analysis. The real-time PCR was carried out using SYBR^®^ Premix Ex Taq™ II (Tli RNaseH Plus) (TaKaRa Biochemicals, China). The 18 S rRNA gene was used to standardize the results by eliminating variation in the quantity and quality. All of the primers used to amplify these genes were designed using software Primer premier 5.0 and the primer pairs are listed in Table 1. The RT-PCR program was: denaturation at 95 °C for 30 s, 40 cycles of 95 °C for 5 s and 60 °C for 30 s, followed by a melt curve program from 60 °C to 95 °C by 0.5 °C increases each 5 s. The relative gene expression abundance was quantified using the 2^−ΔΔCt^ method^34^.

### Protein extraction and immunodetection of protein D1

20 mL algal cultures were centrifuged at 5000 g for 10 min, the pellets were washed twice with PBS solution and then re-suspended in 1 mL PBS solution. The suspension was then sonicated at 100 W for 60 times to lyse the algal cells. The debris was removed at 12000 g for 10 min and the supernatant were transferred to new Eppendorf tubes. The protein concentrations of the supernatant were determined using Bradford assays as mentioned above.

2 μg total protein were separated by SDS-PAGE in a 12% polyacrylamide gel, the resolved protein was then transferred to PVDF membranes and probed with the antibody (Agrisera) raised in rabbits against PSII reaction center protein D1. After incubation with secondary anti-rabbit IgG antibody coupled with horseradish peroxidase for 3 h, the membranes were reacted with ECL solution. Developed films were scanned with an Epson Expression 1680 Scanner^25^.

## Results

### Effects of strain LP-10 on intracellular ROS content of algal cells

**Figure 1:**
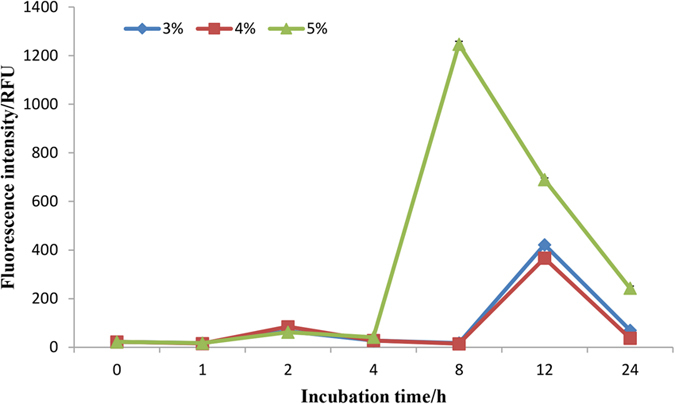
The ROS fluorescence intensity of algal cells under *Bacillus* sp. LP-10 treatment of different concentrations (3, 4, 5%).

The intracellular ROS content of algal cells incubated with 3, 4 and 5% *Bacillus* sp. LP-10 filtrates showed an obvious variation during incubation time (Fig. 1). The fluorescence intensity of algal cells at all treatment concentrations was kept at a low level ( < 100 RFU) in the early, then increased rapidly to a high level in all three treatment concentrations in 8 h (~1200 RFU) or 12 h (~400 RFU), respectively. Subsequently, the ROS levels of all treatments decreased to a low level, but still higher than the initial levels. The results shown in the fluorescence photos of algal cells treated by 5% filtrates were consistent with the fluorescence intensity determined by microplate reader (Fig. S1).

### Effects of ROS scavengers addition on algal cell death

**Figure 2:**
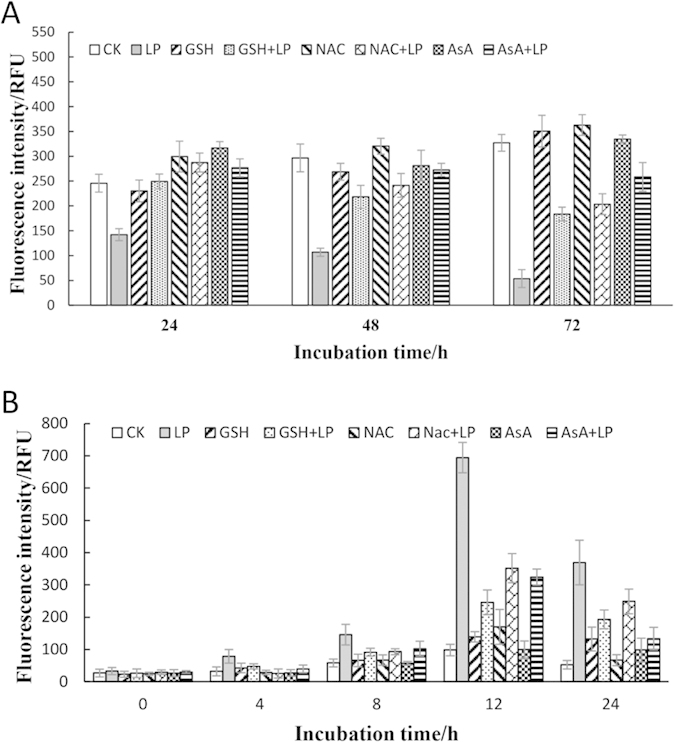
The algal autofluorescence intensity (**A**) and ROS fluorescence intensity (**B**) of algal cells under 5% *Bacillus* sp. LP-10 filtrates treatment with addition of ROS scavengers. CK represents algal cultures without any addition; LP represents algal cultures treated by 5% filtrates of *Bacillus* sp. LP-10; NAC represents algal cultures with addition of NAC; NAC + LP represents algal cultures with addition of NAC treated by 5% filtrates of *Bacillus* sp. LP-10. GSH and AsA are similar with those.

The impacts of addition of ROS scavengers NAC, GSH and AsA on algal cell death are shown in Fig. 2A. The addition of ROS scavengers NAC significantly influenced the algicidal activities of *Bacillus* sp. LP-10 on *P. globosa* cells. The addition of the LP-10 filtrates significantly lower the fluorescence intensity of algal cells, which was obviously less than that of the control (CK). When LP-10 treatment was added with NAC (NAC + LP), the fluorescence intensity of algal cells was much higher than that of the LP-10 treatment without NAC. And the fluorescence intensity of the NAC + LP treatment was approximate to the CK control at 24 h. However, the fluorescence intensity of the NAC + LP treatment was lower than the control at 48 h and 72 h, but still higher than the LP-10 treatment. The trends of addition of scavengers GSH and AsA showed similar pattern with the NAC.

In the ROS scavenger assays, the ROS contents of algal cells in each groups were determined and the results were presented in Fig. 2B. The results indicated that the ROS levels of the treatment groups supplemented with ROS scavengers was apparent lower than the LP-10 treatment. NAC, GSH and AsA could effectively remove the superfluous ROS in the algal cells.

### Effects of strain LP-10 on the MDA content and antioxidant activities of algal cells

The MDA content increased significantly after the algal cells were treated with filtrates (Fig. S2), and the MDA content of the 4 and 5% treatment groups at 72 h were 10.78 and 29.53 times higher than that of the control. The MDA content of the 3% treatment group was also slightly higher than the control at 72 h, which implied that the addition of *Bacillus* sp. LP-10 filtrates promoted the lipid peroxidation of algal cells obviously.

**Figure 3:**
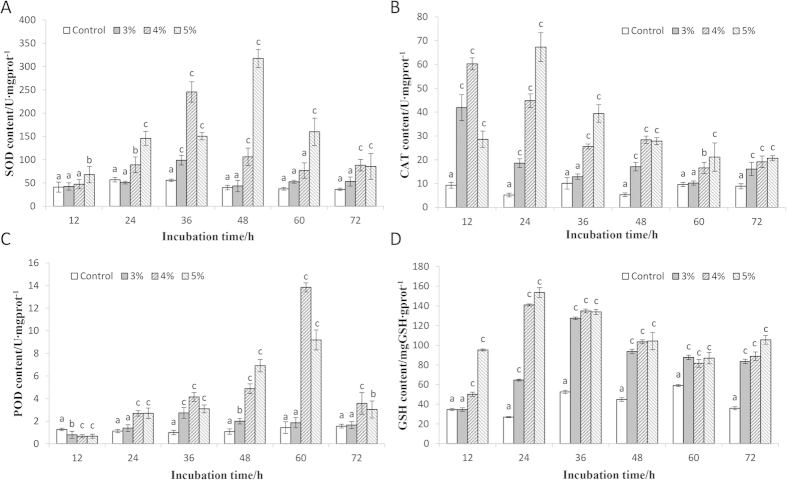
The SOD, CAT, POD and GSH activities of algal cells incubated with LP-10 filtrates of different concentrations (3, 4, and 5%). All data represent the means ± S.D. (**a**) represents no significant difference, (**b**,**c**) represent statistically significant differences of *p* < 0.05 and *p* < 0.01 compared to the control respectively.

SOD activities of the different treatment groups varied based on the concentrations of the LP-10 filtrates (Fig. 3A). All three treatments significantly enhanced SOD activities and the highest SOD activities of the 3, 4 and 5% treatment groups were 1.77-, 4.41- and 7.96-fold of the control. CAT activities shared a similar pattern with the SOD activities but with a rapid increase at the early phase (Fig. 3B). All three treatment groups showed a significant increase of CAT activities after 72 h incubation with LP-10 filtrates. POD activities of algal cells changed slower than CAT activities (Fig. 3C), and the 4 and 5% treatment groups were significant higher than the control. GSH is a type of non-enzymatic antioxidant and plays an important role in protecting cells from oxidative stress. Similarly, the addition of LP-10 filtrates also induced a rapid increase of GSH content in a short time (Fig. 3D), and the GSH content of the three treatment groups was 2.32, 2.47 and 2.93 times higher than that of the control after 72 h treatment.

### Effects of strain LP-10 on chlorophyll content and variable chlorophyll fluorescence

The contents of Chl *a* and *b* significantly decreased after algal cells were incubated with strain *Bacillus* sp. LP-10 filtrates (Fig. S3A,B). With the incubation time prolonged, decline of pigment contents showed the manner of concentration-dependent. The ratio of Chl *a* and Chl *b* were significantly impacted by the LP-10 filtrates (Fig. S3C). In the early phase, the ratio of the two pigments kept stable, but a rapid decrease was observed after the algal cells were incubated with filtrates for more than 36 h, and higher concentration filtrates led to more significant decreases of the ratio. In the late phase, the ratio began to increase and gradually surpassed the control group. These results suggested that the sensitivity of the two pigments to strain LP-10 filtrates was different, and the pigment structures and balance were destroyed by the filtrates.

The photosynthetic efficiency measured from maximum quantum yields (Fv/Fm) showed an apparent decrease after the algal cells were treated with LP-10 filtrates. Higher concentration filtrates and longer treatment time led to more severe damage to the algal cells (Fig. S4A). The Fv/Fm value of the 5% treatment group at 48 h was approximate to 0.3, far less than the normal level. The maximum rETR, which was used to evaluate the maximum photosynthetic capacity, showed a similar change pattern with Fv/Fm (Fig. S4B) and the rETR of all three treatment groups significantly declined after the algal cells were treated with strain LP-10 filtrates.

### Effects of light conditions on algal cells

**Figure 4:**
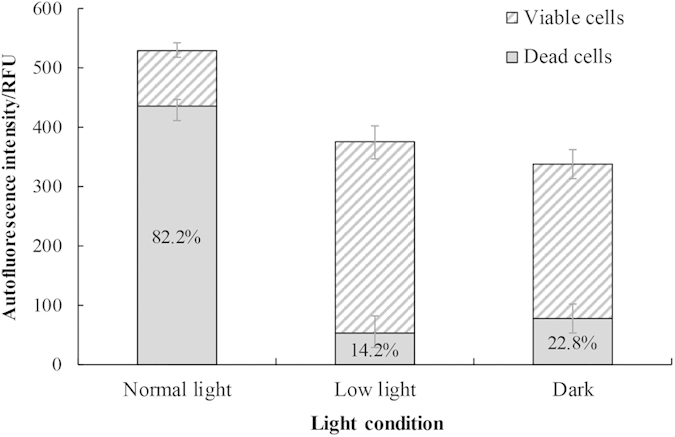
The autofluorescence intensity of algal cells under different light conditions after the algal cells were incubated with 5% LP-10 filtrates for 72 h. Normal light, low light and dark represent light intensities of 50, 20 and 0 μmol photons m^−2^s^−1^. The whole bar represents autofluorescence intensity of the control, while the twill part of the bar represents algal autofluorescence intensity of the 5% LP-10 treatment. And the gray part of the bar represents difference between the treatments and control, namely the dead cells under each light conditions.

The effects of different light conditions on the algicidal activities of strain LP-10 were presented in Fig. 4. The proportion of dead cells varied based on the light conditions. The algicidal activities of 5% LP-10 filtrates treatment under normal light were 82.2% at 72 h, while those of the low light and dark treatment groups were 14.2% and 22.8% respectively, which were significantly lower than that of the normal light treatment group.

### Effects of strain LP-10 on the gene expression of algal cells

**Figure 5:**
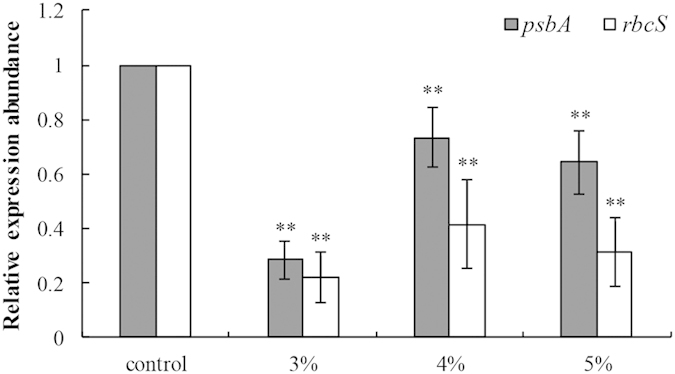
The relative transcriptional expression abundance of two genes (*psbA* and *rbcS*) in algal cells after incubation with LP-10 filtrates for 24 h. All data represent the means ± S.D. **represents statistical significance at the *p* < 0.01 level.

Genes *psbA* and *rbcS* play important roles in the photosynthesis of plant and algal cells. Gene *psbA* is responsible for the PSII reaction center protein D1, and gene *rbcS* codes for the small subunit of Rubisco, an important enzyme for CO_2_ fixation. Our results demonstrated that the transcriptional expression abundance of *psbA* was 0.29, 0.73 and 0.64-fold of that of control at 24 h when treated by LP-10 filtrates (Fig. 5). And the transcriptional abundance of *rbcS* at 24 h shared a similar pattern with the *psbA* and all three concentrations treatments suppressed the gene expression of *rbcS*.

### Effects of strain LP-10 on the expression of PSII reaction center protein D1

**Figure 6:**
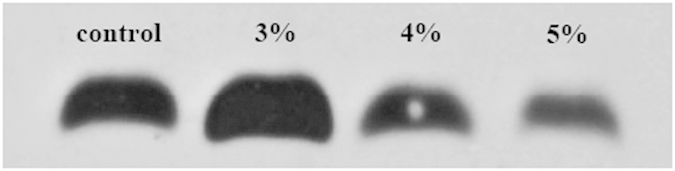
Immunodetection of the PSII reaction center protein D1 after the algal cells were incubated with LP-10 filtrates for 24 h. Control represents the algal cells without addition of LP-10; 3, 4, 5% represent the algal cells treated by different concentrations of LP-10 filtrates respectively.

The expression of PSII reaction center protein D1 was detected in this study (Fig. 6). The D1 content of the 3% treatment group was higher than that of the control while the content of the 4 and 5% treatment groups was remarkably decreased. The D1 content of the 3, 4 and 5% treatment groups was 169, 71 and 43% of the control, based on the quantification of band intensities using densitometry with Quantity-One software.

## Discussion

Marine disasters bring about huge loses to human every year, and red tides was one of the most notorious calamities. However, ocean also provide huge resources for us as a library which make it possible to utilize marine microorganisms to regulate the HABs. Marine algicidal bacteria are suggested to be excellent candidates in managing HABs^8^,^10^. Up to now, many bacteria have been isolated to be effective in removing harmful algae, but the action mechanism of algicidal bacteria on toxic algae has not been well explained. In this study, we investigated the mode of action of a marine algicidal bacterium *Bacillus* sp. LP-10, which was previously isolated in our laboratory from the surface seawater samples of the East China Sea.

As is known, ROS are common byproducts of aerobic metabolism, predominantly produced in chloroplasts and mitochondria of algal cells^19^. Normally, antioxidants system of algal cells could remove the superfluous ROS to keep the cells from oxidative damage. However, environmental stresses often induce algal cells an unusual rise of ROS level. Liu *et al.* found the addition of allelochemical florfenicol significantly enhanced the intracellular ROS content of *Skeletonema costatum* at 6 h^35^. Similarly, an obvious increase of ROS was observed in our study when strain LP-10 was inoculated into the algal cultures, which indicated that the algal cells were suffering from oxidative stress. And the MDA content rise also confirmed the oxidative damage in the algal cells.

NAC is a commonly used ROS scavenger, and our results demonstrated that the addition of NAC to the algal cultures significantly improved the survival rate of algal cells compared to algal cells treated by LP-10 filtrates without NAC. And the ROS content determination in ROS scavenger assays hinted that the superfluous ROS was really removed by the ROS scavengers. The tested results of two other antioxidant (GSH and AsA) showed similar consequences and confirmed that the superfluous ROS was reacted with the scavengers rather than surviving the algal cells by other means. Furthermore, the autofluorescence intensity of algal cells in NAC + LP treatment group was approximate to the CK and NAC control groups, suggesting that the ROS was the main reason for algal cell death.

MDA content was an indicator of lipid peroxidation in algal cells. Our results suggested the MDA content were keeping rising during the experiment time, and the rate of lipid peroxidation became more and more serious, implying the antioxidant system of algal cells may not clear the excessive ROS in time and the algal cells suffered from oxidative damage for long. Other reported isolates were also proved to cause the increase of MDA content in algal cells^15^,^36^.

Algal cells execute programs to clear the excess ROS via antioxidant systems^21^. Our results showed that addition of LP-10 enhanced SOD, CAT, POD and GSH activities of algal cells, the activities of these four antioxidants at 72 h were significantly higher than the initial levels, suggesting algal cells were kept under the oxidative stress and superfluous ROS could not be cleared in time to resume the normal status. Excess ROS triggered a series of damage processes in algal cells^22^, leading the dysfunction of many fundamental organelles and structures. MDA assays also revealed that algal cells were under continuous oxidative stress as discussed above, which will oxidize the lipids of membranes to MDA, thus the membrane permeability changed, and the algal cells finally disrupted for dysfunction of algal cells.

Environmental factors often affected important physiological functions of algal cells^23^,^26^. Domingues *et al.* investigated the response of *Phaeodactylum tricornutum* to high light exposure, the results showed photosynthetic activities of algal cells declined obviously^25^. In this study, addition of *Bacillus* sp. LP-10 filtrates significantly impacted the fundamental functions of algal cells. Among which, photosynthesis systems are vulnerable to oxidative stress. Photosynthesis pigments are key components of photosynthesis systems, which are responsible for light harvesting and photosynthetic reaction^37^. When LP-10 filtrates was inoculated into the algal cultures, an obvious decrease of Chl *a* and *b* contents was observed, and the ratio of these two pigments was significantly changed, suggesting the pigment structures were destroyed, scanning electron microscope (SEM) and transmission electron microscope (TEM) images of algal cells under treatment also confirmed the results (Figs S5, S6). The data of electron microscope indicated the thylakoid of the algal cells became sparse and structure of the chloroplast structures were changed. The unstable ratio of Chl *a* and *b* contents may be the consequence of sensitivity difference of these two pigments under strain LP-10 stress. The low ratio in the early phase indicates that photosynthetic antenna complexes are more vulnerable to oxidative stress than light-harvesting complexes^17^.

The Fv/Fm and maximum rETR are two important parameters used to evaluate the photosynthesis efficiency and capacity^38^, which were usually disturbed by environmental stresses. Domingues *et al.* found high light exposure will lead declination of the Fv/Fm of algal cells^25^. Our results revealed that the value of Fv/Fm significantly decreased when the algal cells were under strain LP-10 stress, suggesting the photosynthetic efficiency was hindered by strain LP-10. Similarly, the rETR was also significantly inhibited by strain LP-10, implying that the photosynthetic capacity of algal cells decreased after treatment with strain LP-10. These results indicated that the photosynthesis systems of algal cells were attacked by the active metabolites produced by *Bacillus* sp. LP-10. Combined with the pigment assays, we speculated that strain LP-10 would destroy the structures of photosynthetic system and lower the photosynthesis activities, causing the dysfunction of the photosynthetic system at last.

More than that, *Bacillus* sp. LP-10 also inhibited the transcriptional expression of many important genes. Wu *et al.* noted that allelochemical pyrogallic acid significantly affects the expression of genes *psbA*, *grpE*, *fabZ*, *recA*, *cmpA*, *ftsZ* and *cyrJ*^39^. In our study, two photosynthesis-related genes (*psbA* and *rbcS*) were investigated. At 24 h, transcriptional expression of both gene *psbA* and *rbcS* were down-regulated. We speculated that the active metabolites produced by strain LP-10 may affected the transcription processes or severe impairment occurred in the genetic molecules of the cells at the time. Gene *psbA* was responsible for synthesis of protein D1, one of the subunits of the reaction center of PSII. Impairment of protein D1 will decrease the photosynthetic capacity, leading to the photoinhibition of the algal cells^24^. The gene *rbcS* code for the small subunit of Rubisco, which plays a critical role in CO_2_ fixation and photorespiration^40^. The inhibition of the *rbcS* in the algal cells implied that the CO_2_ fixation cycle (Calvin cycle) was interrupted by *Bacillus* sp. LP-10, which would accelerate the photoinhibition of the algal cells^41^, because limitation of CO_2_ fixation will decrease the absorption capacity for solar energy of the algal cells and affect the electron transportation of photosystems. The excess energy causes the production of numerous ROS at last, which inhibits the synthesis of protein D1^23^.

Our study also found that light was an indispensable factor in algal cell death. The algicidal activity of strain LP-10 under low light was very low (~15%) while normal light led to a higher algicidal activity (~80%), which meant the light intensity of “normal light” was too high for the stressed algal cells and the stressed algal cells could not utilize the “normal light”, so that the relative high light leads to photoinhibition of the algal cells, which indirectly revealed that strain LP-10 impacted the CO_2_ fixation and the utilization of solar energy. The results implied that dysfunction of the photosynthesis system (photoinhibition) was the most fundamental reason for algal cell death. Qian *et al.* and Yang *et al.* reported that the allelochemicals N-phenyl-2-naphthylamine and hydroquinone could cause photoinhibition of *Chlorella vulgaris* and *Phaeodactylum tricornutum*^17^,^18^. Though some other reasons were able to cause the algal cell death, such as destroying the cell structures, our results demonstrated the role of other factors were minor reasons. Because when the algal cultures were placed in the dark or under low light conditions, the algicidal rate was very low (~15%).

The western blot analysis of D1 protein showed that the D1 protein content of algal cells at 4 and 5% treatment concentrations of strain LP-10 decreased obviously at 24 h. This results were consistent with the gene expression profiles of *psbA*, which confirmed that photoinhibition was taken place in the algal cells. Photoinhibition of algal cells indicated the photosynthesis would be hampered, and energy-consuming functions would be inhibited. Long-time photohibition would be unable to support the survival of the algal cells, leading to the algal cells death eventually.

**Figure 7:**
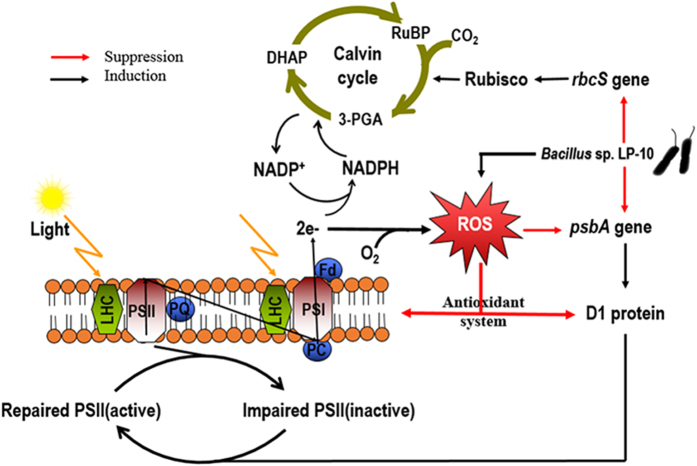
A hypothetical scheme for the algicidal mechanism of the algal cells upon *Bacillus* sp. LP-10.

From the above, we conclude that algicidal bacterium *Bacillus* sp. LP-10 induce a significant increase of ROS in algal cells, and continuous stressing make the superfluous ROS not being cleared in time, which disturb the physiological functions and destruct the structures of algal cells. More important, excess ROS lead to photoinhibiton of algal cells by influencing the expression of the photosynthesis-related genes and protein, and impaired photosystem apparatus cannot be repaired, leading to the dysfunction and causing disruption of algal cells eventually (Fig. 7).

## Additional Information

**How to cite this article**: Guan, C. *et al.* Photoinhibition of *Phaeocystis globosa* resulting from oxidative stress induced by a marine algicidal bacterium *Bacillus* sp. LP-10. *Sci. Rep.* **5**, 17002; doi: 10.1038/srep17002 (2015).

## Supplementary Material

Supplementary Information

## Figures and Tables

**Table 1:** The sequences of the primer pairs for RT-PCR analysis.

Gene name	Forward primer (5′–3′)	Reverse primer (5′–3′)
18 S	TCCGATAACGAACGAGAC	TGACGCAAACTTCCACTT
*psbA*	AGTTGCTGGTTCTCTACTTTACG	TTCCCACTCACGACCGATG
*rbcS*	AAGTCTTACTGGGAAATGTGGG	AGCAGGACGCTGAACGATG
