# Supplementary material for: Photoinhibition of Phaeocystis globosa resulting from oxidative stress induced by a marine algicidal bacterium Bacillus sp. LP-10
Source: Sci Rep. 2015 Nov 25;5:17002. doi: 10.1038/srep17002 (PMC4658505; doi:10.1038/srep17002)
Supplement: Supplementary Information [file srep17002-s1.pdf]

Supplementary Information

Photoinhibition of *Phaeocystis globosa* resulting from oxidative stress induced by a marine algicidal bacterium *Bacillus* sp. LP-10

Guan Chengwei[1,2※], Guo Xiaoyun[1, 3※], Li Yi[1,4], Zhang Huajun[1], Lei Xueqian[1], Cai Guanjing[1], Guo Jiajia[1], Zhiming Yu[5]*, Zheng Tianling[1]*

1, State Key Laboratory of Marine Environmental Science and Key Laboratory of Ministry of Education for Coastal and Wetland Ecosystems, School of Life Sciences, Xiamen University, Xiamen 361102, China.

2, Tobacco Science Research Institute of Jiangxi Province, Nanchang 330025, China.

3, School of Chemistry and Chemical Engineering, Xiamen University, Xiamen 361102, China.

4, College of Life Sciences, Henan Normal University, Xinxiang 453007, China

5, Key Laboratory of Marine Ecology and Environmental Science, Institute of Oceanology, Chinese Academy of Sciences, Qingdao 266071, China.

※ These two authors contributed equally to this work.

*Corresponding authors: Zheng Tianling, E-mail: wshwzh@xmu.edu.cn; Yu Zhiming, E-mail: zyu@ms.qdiao.ac.cn.

T1 The toxicity test of the *Bacillus* sp. LP-10

The toxicity tests followed the manufacture instructions of the Microtox® with slight modifications.

The luminescent bacterium *Photobacterium phosphoreum* was stored at -80 ℃ and activated with bouillon peptone medium containing 3% NaCl and 1% glycerin in a rotary shaker (28 ℃, 200 rpm) for 18-24h. To check whether the bacterium is available for toxicity test, 5μL of the culture was added to 1.5mL fresh 2216E medium and the relative luminosity (RLU) was measured with a Berthold Sirius L Luminometer. The RLU remained relatively stable between 5,000,000 and 6,000,000 for 20 min, indicating *P. phosphoreum* used in the following bioluminescent assay was the standard growth status.

For toxicity tests, *Bacillus* sp. LP-10 was cultured for different time. The filtrates of strain LP-10 for different culture time (1-7d) were prepared as mentioned in the manuscript. During the experiments, each experimental tube, which contained 1.5mL filtrates of *Bacillus* sp. LP-10, was coupled with a control tube containing the same 1.5mL fresh 2216E media. 5μL of the *P. phosphoreum* culture was added to each tube and measured for the RLU after a precisely 15min reaction. The inhibition ratio of the luminosity was calculated using the following formula:

Relative luminosity (%) $= \frac{\mathrm{Lt}}{\mathrm{L0}} \times 100$,

where Lt is the luminosity of the experimental tube, and L_0 the luminosity of the control tube.

All experimental groups were replicated for three times.

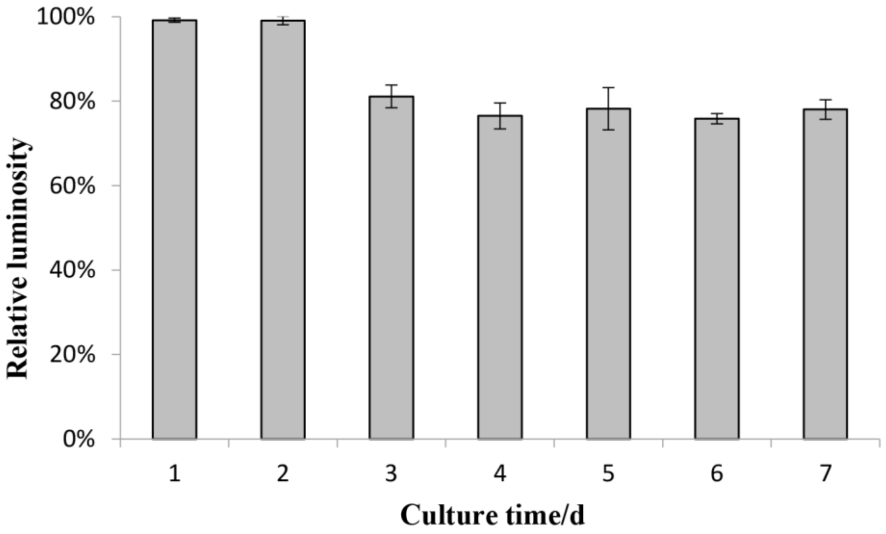

Fig. T1 The relative luminosity of *P. phosphoreum* incubated with *Bacillus* sp.LP-10 filtrates of different culture days.

Results

The toxicity tests were performed to check the ecological safety (Fig. T1). The filtrates of *Bacillus* sp.LP-10 for different culture days were incubated with the luminescent bacterium *P. phosphoreum* and the relative luminosity were determined. If the strain was toxic, the luminosity of the luminescent bacterium *P. phosphoreum* would be inhibited and the relative luminosity is less than 100%. Generally, if the relative luminosity of the tested organisms is more than 70%, they are considered as nontoxic species or slight toxic species, which are safe for the environment. In our results, the relative luminosity of the filtrates for 7 different culture time were higher than 75% (76-99%), indicating that the algicidal bacterium is safe for the environment.

Supplemtary Figures

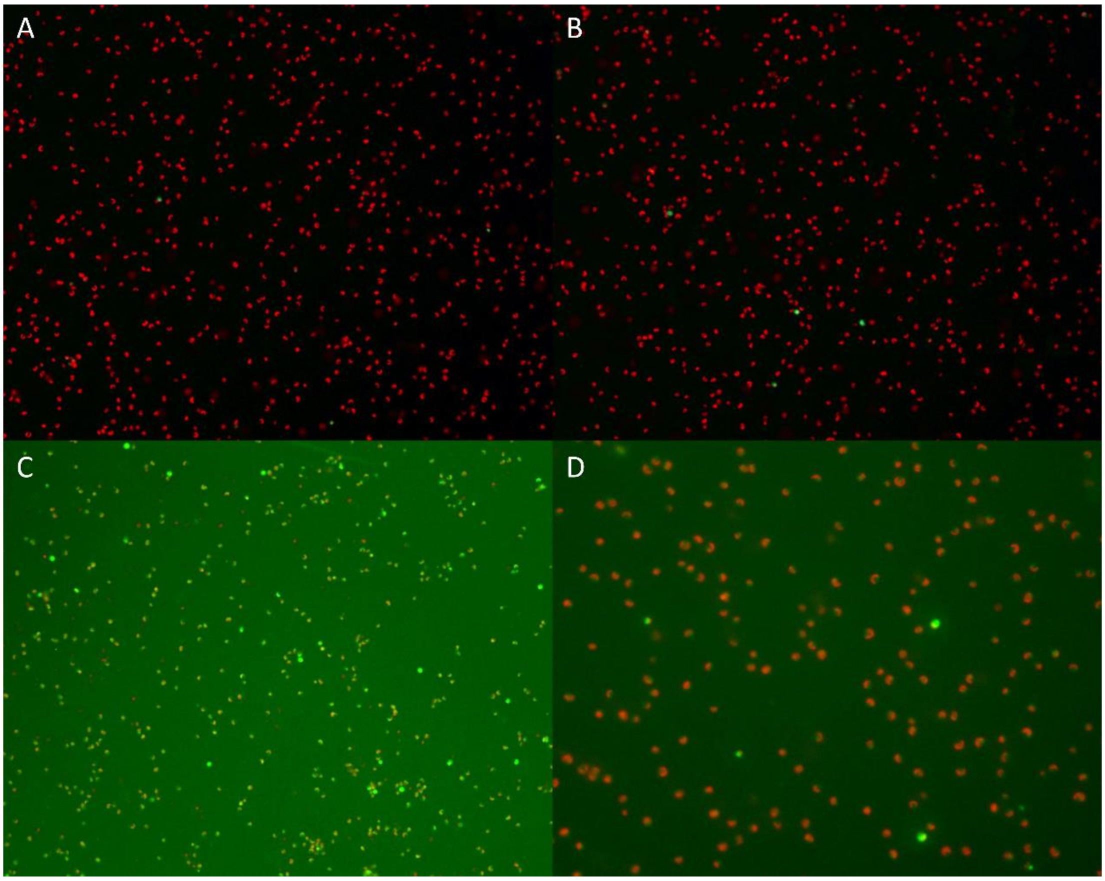

Fig. S1 The fluorescence microscope images of algal cells treated by 5% *Bacillus* sp. LP-10 filtrates. Letter A, B, C, D represent the images taken at 0, 2, 8 and 12h after treated by 5% LP-10 filtrates respectively.

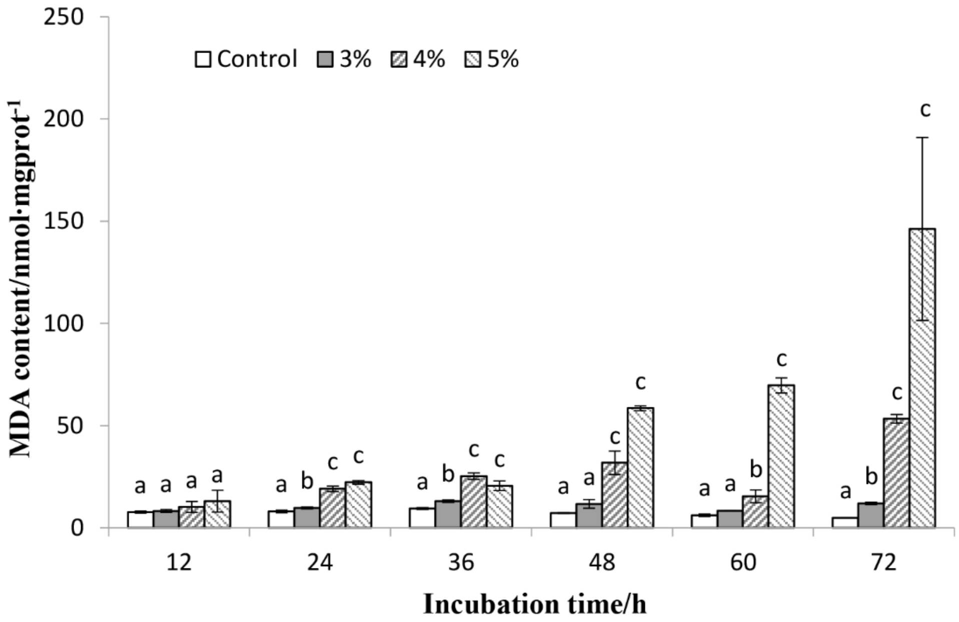

Fig. S2 The MDA content of algal cells incubated with *Bacillus* sp. LP-10 filtrates. All data represent the means ± S.D. **a** represents no significant difference, **b** and **c** represent statistically significant differences of $p < 0.05$ and $p < 0.01$ compared to the control.

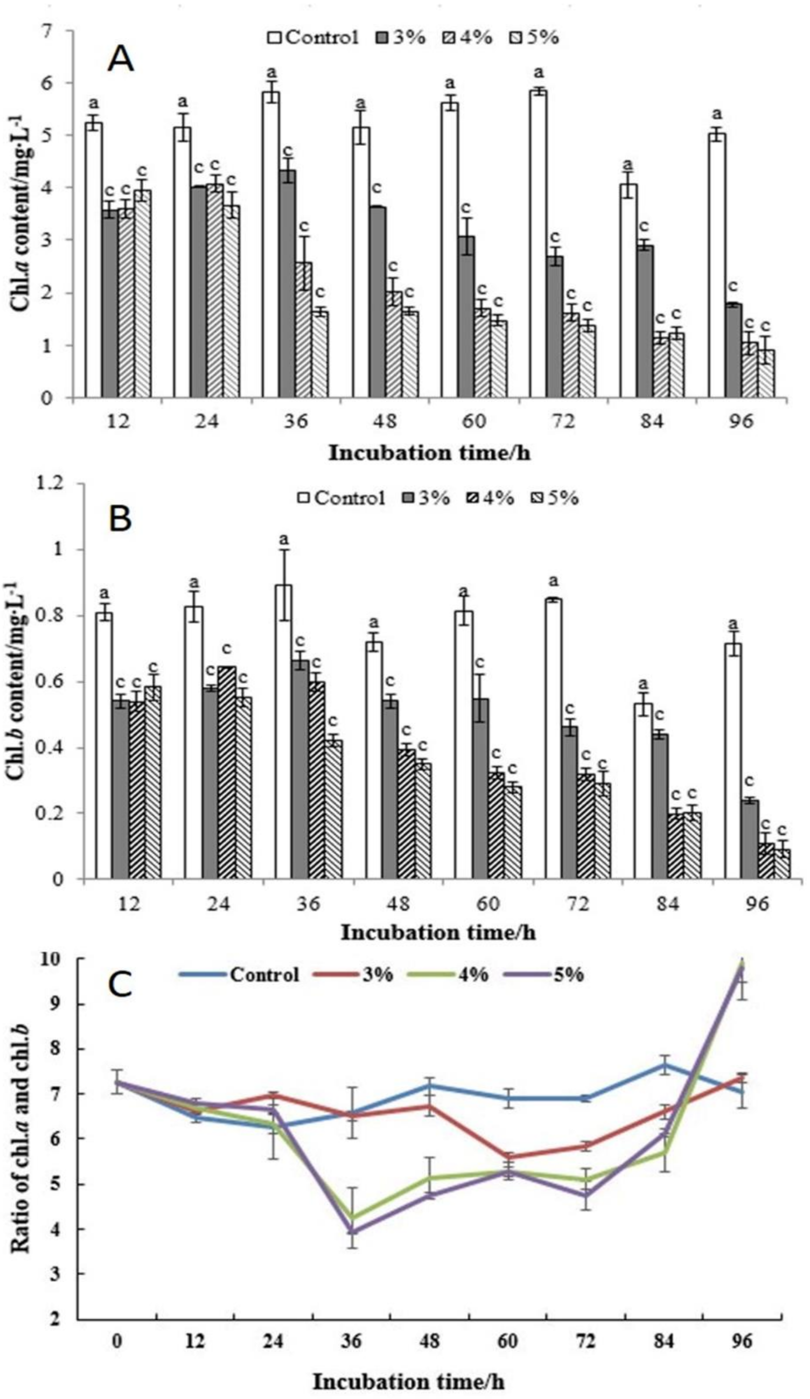

Fig. S3 The Chl *a* (A) and *b* (B) content and the ratio of Chl *a* to Chl *b* (C) in response to *Bacillus* sp. LP-10 filtrates. All data represent the means ± S.D. **a** represents no significant difference, **b** and **c** represent statistically significant differences of $p < 0.05$ and $p < 0.01$ compared to the control.

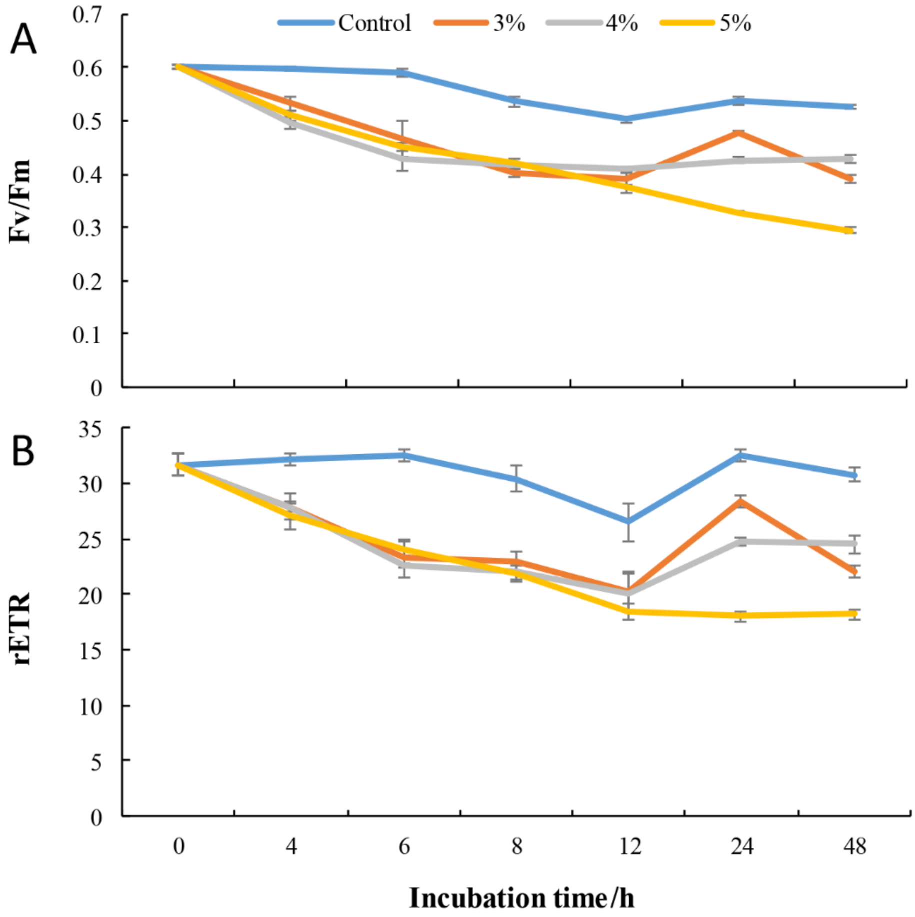

Fig. S4 The maximum quantum yields (Fv/Fm) (**A**) and maximum relative electron transport rate (rETR) (**B**) of algal cells after incubation with *Bacillus* sp. LP-10 filtrates. All data represent the means ± S.D.

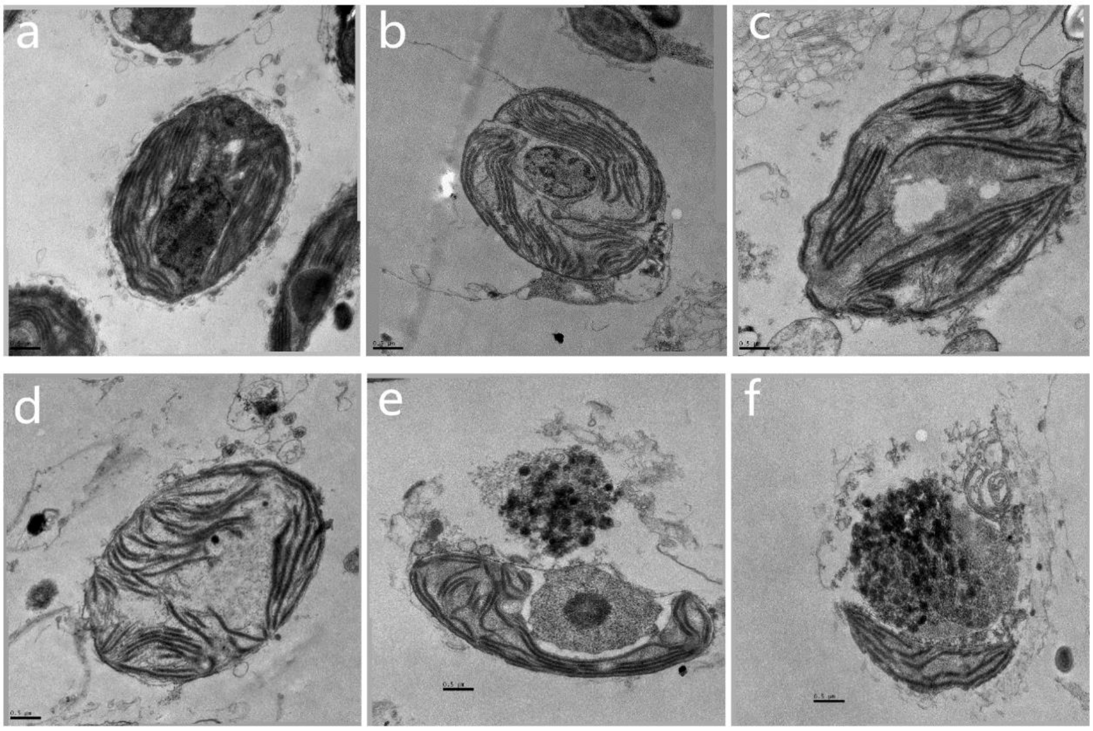

Fig. S5 The transmission electron microscope (TEM) images of algal cells treated by 5% *Bacillus* sp. LP-10 filtrates. The letter **a**, **b**, **c**, **d**, **e**, **f** represents image of treated algal cells at 0, 12, 24, 36, 48, 60, 72h, respectively.

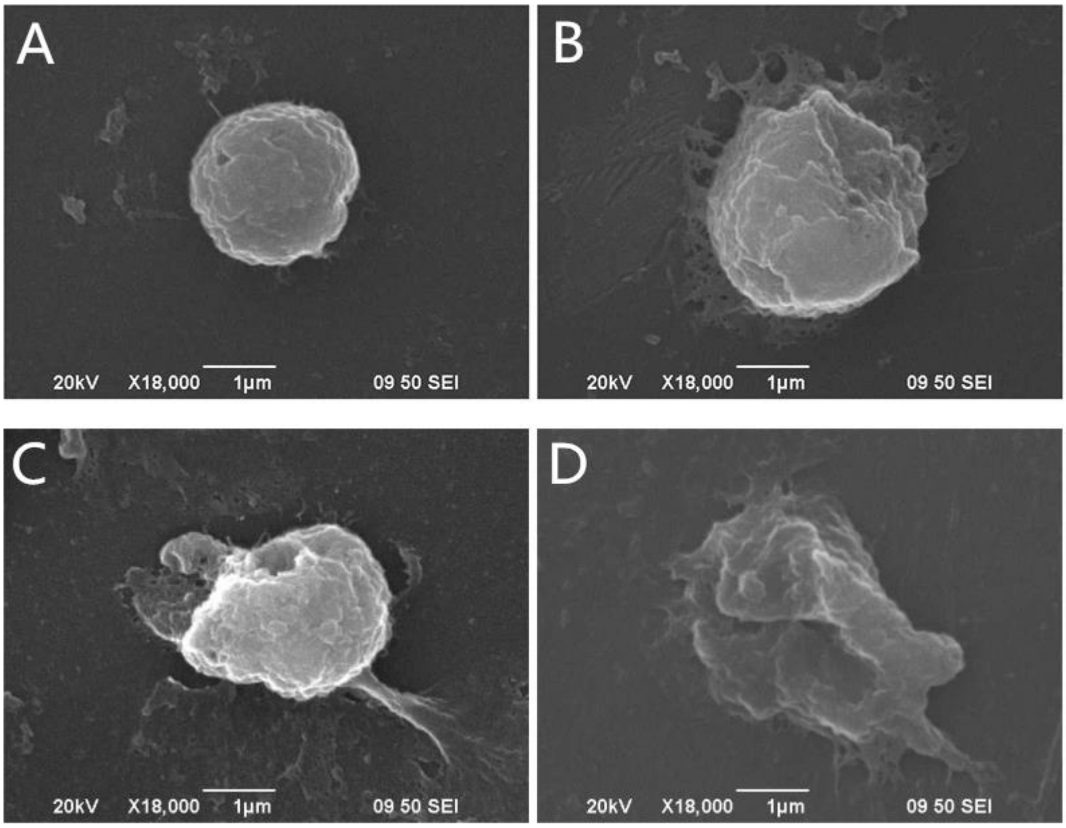

Fig. S6 The scanning electron microscope (SEM) images of algal cells treated by 5% *Bacillus* sp. LP-10 filtrates. The letter **A**, **B**, **C**, **D** represents image of treated algal cells at 0, 24, 48, 72h, respectively.

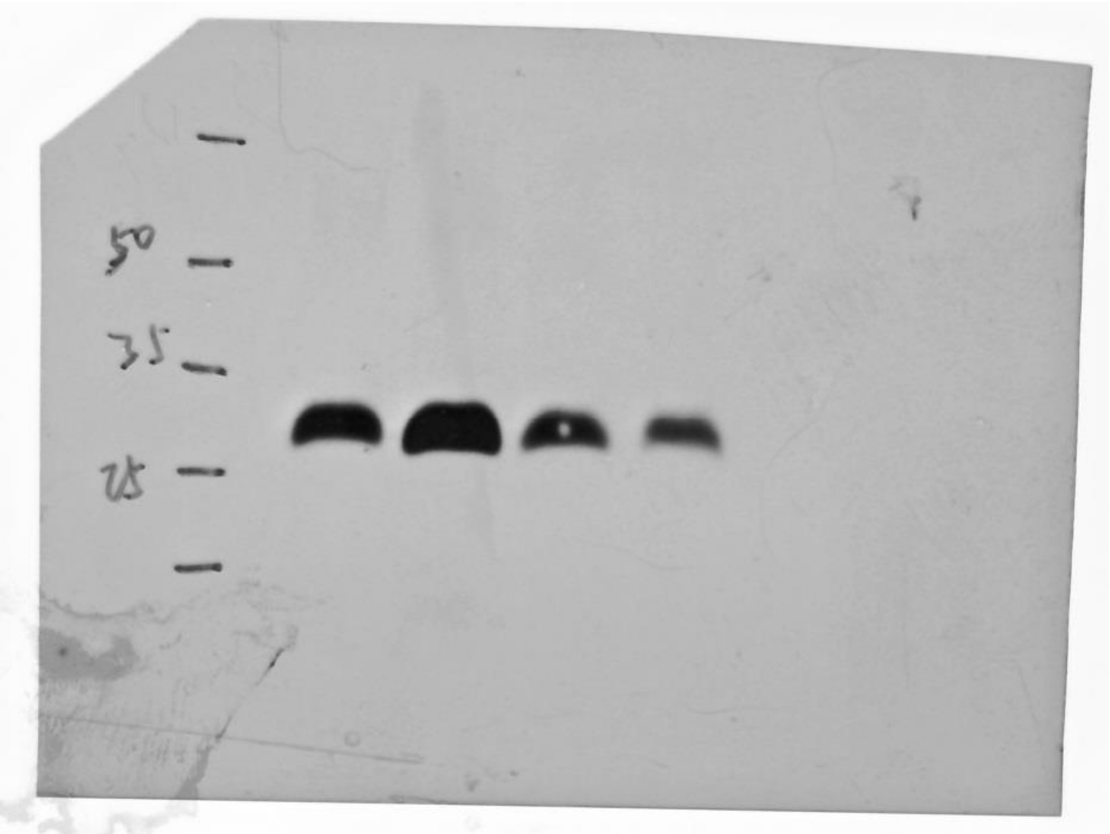

Fig. E1 The original image of **Fig. 6** Immunodetection of the PSII reaction center protein D1 after the algal cells were incubated with LP-10 for 24h. From left to right, four lanes represent different treatments of **control, 3, 4, 5%**, respectively.
